# Urban noise-induced stress and decision-making: effects of high-intensity acoustic exposure on risk-based and intertemporal choices

**DOI:** 10.3389/fpsyg.2026.1899506

**Published:** 2026-08-10

**Authors:** Jorge Alexander Ríos-Flórez, Leidy Parra Restrepo, David Santiago Vivares-Serna

**Affiliations:** 1 Psychology Laboratory, Department of Psychology, Politécnico Grancolombiano University Institution, Medellín, Colombia; 2 Hippocampus Research Group and SAPIENS: Neurosciences and Behavior, Medellín, Colombia

**Keywords:** autonomic nervous system, decision-making, delay discounting, heart rate, noise, prefrontal cortex, psychological stress – [term MeSH], risk-taking

## Abstract

**Introduction:**

Urban noise is one of the most pervasive environmental stressors in Latin American cities, yet its specific effects on complex cognitive processes such as decision-making remain insufficiently studied. This study examined the effects of acute exposure to high-intensity urban noise (85–95 dB[A]) on risk-based and intertemporal decision-making in healthy adults under controlled laboratory conditions, while characterizing the autonomic and subjective stress response and exploring its relationship with cognitive performance.

**Methods:**

A quasi-experimental between-groups design was employed with *N* = 134 university students (Experimental Group *n* = 56; Control Group *n* = 78), equivalent in age, sex, years of schooling, baseline heart rate, and perceived stress. Participants completed the Iowa Gambling Task (IGT) and the Monetary Choice Questionnaire (MCQ) under noise or silence conditions. Heart rate was recorded at six time points across the protocol, and perceived stress was assessed using the PSS-14 and a visual analogue scale.

**Results:**

The Experimental Group showed significantly elevated heart rate across all experimental phases (*d* = 2.50 for ΔHR) and markedly higher post-task stress perception (*r* = 0.856), confirming the efficacy of the experimental manipulation. However, no significant between-group differences were found in IGT performance or MCQ delay discounting parameter log(*k*), a pattern that remained after controlling for baseline physiological and psychological variables (ANCOVA) and was confirmed by mediation analyses showing that ΔHR did not mediate the relationship between experimental condition and decision-making performance.

**Discussion:**

These findings reveal a systematic dissociation between physiological stress activation and cognitive performance, suggesting that autonomic sympathetic arousal induced by urban noise is not a sufficient condition to impair prefrontal-dependent decision-making processes under acute exposure conditions.

## 1 Introduction

Human decision-making rarely occurs under neutral or ideally controlled conditions. In everyday life, people must make consequential decisions—personal, economic, professional, and social—in environments characterized by significant levels of sensory stimulation and concurrent cognitive demands. Among the most pervasive and underestimated environmental stressors is urban acoustic noise. The progressive growth of cities worldwide has intensified exposure to high-intensity noise generated by vehicular traffic, commercial activity, industrial operations, and human concentrations. This acoustic landscape constitutes not merely a nuisance, but a recognized public health problem with documented neurobiological, cardiovascular, and behavioral consequences (Münzel et al., 2025; World Health Organization, 2018).

The WHO Environmental Noise Guidelines for the European Region (World Health Organization, 2018) established that exposure to road traffic noise above 53 dB(A) is associated with measurable adverse health effects, and more than 30% of Europeans—approximately 150 million people—are chronically exposed to levels exceeding this threshold (Münzel et al., 2026). This problem is not limited to the European context: in Brazil, levels consistently exceeding 55 dB(A) have been documented in residential areas and main roads (Paiva et al., 2019; Zannin et al., 2003), and in Mexico values reaching 96 dB(A) have been recorded in high-activity commercial zones (Zamorano-González et al., 2021)—comparable to those employed in the present study. The noise intensities characteristic of high-density urban environments (85–95 dB[A]) constitute a potent situational stressor with immediate psychophysiological consequences exceeding the thresholds identified by the WHO as the limit for observable health effects (Münzel et al., 2018; World Health Organization, 2018). The noise burden in Latin American cities thus represents an underaddressed public health risk, with implications extending beyond auditory damage to include effects on the autonomic nervous system, psychological wellbeing, and, as will be argued in this paper, cognition, and decision-making behavior (Münzel et al., 2020; World Health Organization, 2018).

The growing global concern over environmental noise has spurred significant advances in urban noise assessment, monitoring, and management. Noise distribution within cities is fundamentally shaped by urban design factors—including street morphology, land use patterns, traffic density, and building configurations—all of which modulate acoustic exposure across residential, commercial, and industrial zones (Barrigón et al., 2018). Recent methodological developments in noise monitoring have incorporated machine learning approaches, including artificial neural networks (ANN) and multiple linear regression models, to predict traffic-induced noise levels with accuracy rates exceeding 90% (*R*^2^ = 0.93), offering increasingly precise tools for sustainable urban planning and acoustic risk management (Patel et al., 2024; Tiwari et al., 2024).

The biological pathway through which noise induces stress involves two primary neuroendocrine systems: the sympathetic-adrenal-medullary (SAM) axis and the hypothalamic–pituitary–adrenal (HPA) axis. The SAM response is characterized by the immediate release of catecholamines—epinephrine and norepinephrine—generating autonomic activation, increased heart rate, and elevated blood pressure (Münzel et al., 2018). The HPA axis, activated subsequently, culminates in cortisol secretion by the adrenal cortex following the sequential release of CRH in the hypothalamus and ACTH in the anterior pituitary (Kirschbaum and Hellhammer, 1994). Cortisol exerts broad genomic and non-genomic effects on the central nervous system, modulating neuronal excitability, synaptic plasticity, and executive function (Arnsten, 2009). A systematic review of pharmacological interventions targeting both axes confirmed that differential activation of the HPA axis and the SAM system produces dissociable effects across distinct decision-making domains (Sarmiento et al., 2024a).

Beyond neuroendocrine activation, the health consequences of environmental noise exposure extend across multiple physiological and psychological systems. Chronic urban noise exposure has been associated with disruption of circadian rhythms and hormonal regulation, including melatonin suppression, elevated evening cortisol, altered glucose-insulin profiles, and increased inflammatory markers (C-reactive protein, IL-6), conferring heightened risk for cardiovascular disease, type 2 diabetes, and depression (Badea, 2025). A recent systematic review and meta-analysis synthesizing 11 studies (*N* = 15,525 adults) reported a pooled relative risk of RR = 1.29 (95% CI: 1.16–1.51) for depression and anxiety symptoms associated with chronic environmental noise exposure, with road traffic noise identified as the primary exposure source (Hu, 2025). Epidemiological evidence further indicates that each 10 dB(A) increase in traffic noise is associated with elevated odds of depression (OR = 1.04) and anxiety (OR = 1.12), with environmental noise estimated to account for 1.6 million disability-adjusted life years (DALYs) lost annually in Europe alone (Hahad et al., 2025). At the individual level, noise-induced annoyance has been identified as the strongest predictor of subjective health complaints (*β* = 0.609), operating as a primary mediating pathway between acoustic exposure and adverse health outcomes including anxiety, sleep disturbance, and headache (Tiwari et al., 2023). These convergent findings underscore that the health burden of urban noise pollution is not limited to auditory damage, but encompasses a broad spectrum of systemic effects directly relevant to cognitive and behavioral functioning.

Of relevance to cognitive performance is the sensitivity of the prefrontal cortex (PFC) to stress-induced neurochemical changes. Arnsten (2009) demonstrated that even mild, uncontrollable acute stress produces a rapid and dramatic loss of PFC-dependent cognitive abilities—working memory, inhibitory control, attentional flexibility, and planning—, mediated by elevated catecholaminergic stimulation that activates intracellular signaling cascades (protein kinase C and cyclic AMP–protein kinase A) that weaken synaptic efficacy in dendritic spines. Consequently, the brain shifts from deliberative, goal-directed processing toward habitual processing governed by the amygdala and striatum (Arnsten, 2009, 2015), compromising higher-order cognition including complex decision-making. The neural substrates of decision-making are themselves highly susceptible to this shift: Bechara and Damasio (2005) proposed that adaptive decision-making under uncertainty critically depends on bodily and emotional signals—somatic markers—processed in the ventromedial PFC and amygdala, whose circuit substantially overlaps with the one most vulnerable to stress-induced dysfunction.

At the cardiac level, noise-induced autonomic activation is reflected in measurable changes in heart rate and heart rate variability (HRV), indices of autonomic nervous system balance widely validated as objective indicators of stress and cognitive load (Immanuel et al., 2023; Kim et al., 2018). Crucially, HRV has been directly linked to decision-making performance: Forte et al. (2022) found, in a systematic review of 15 studies with 1,051 healthy participants, that higher vagally mediated HRV was consistently associated with better performance in risk-based decision-making, a result consistent with the Neurovisceral Integration Model (Thayer and Lane, 2000) and with evidence that good decision-makers exhibit higher vagal HRV both at rest and during task performance (Arechavala et al., 2023; Forte et al., 2019).

Decision-making can be classified into two domains particularly sensitive to stressor-induced alterations. The first is risk-based decision-making, involving the evaluation of alternatives with known or estimable outcome probabilities. The second is intertemporal decision-making, concerning choices between outcomes that vary in temporal proximity and magnitude, operationalized through delay discounting—the tendency to devalue rewards as a function of the time required to receive them (Frederick et al., 2002). Both domains involve prefrontal-subcortical circuits implicated in reward valuation, impulse control, and prospective cognition, and both have been shown to be disrupted by psychological stress (Duque et al., 2022; Starcke and Brand, 2016).

Experimental evidence supports that acute stress compromises both domains. Starcke and Brand (2016) synthesized data from 32 experimental datasets (*N* = 1,829) and demonstrated that stress produces more disadvantageous and risky decisions, with a moderate effect size (*d* = 0.26), through stress-induced increases in dopaminergic firing in reward circuits and reductions in prefrontal executive control; the IGT emerges as one of the most sensitive paradigms to these effects given its dependence on the ventromedial PFC (Starcke and Brand, 2012). The systematic review by Duque et al. (2022), encompassing 18 studies with cortisol measurement, confirmed that effect magnitude is modulated by stressor type, individual factors, and the timing of assessment relative to the hormonal peak. In the intertemporal domain, Riis-Vestergaard et al. (2018) and Kimura et al. (2013) demonstrated that hydrocortisone and acute psychosocial stress increase delay discounting, particularly in cortisol responders, although some studies report null effects under certain protocols (Haushofer et al., 2013). The integrative review by Sarmiento et al. (2024b) reconciles these inconsistencies by highlighting the role of stressor type, timing of assessment, and individual differences in physiological reactivity, findings convergent with those reported in a systematic review of pharmacological interventions on the HPA and SAM axes (Sarmiento et al., 2024a).

Despite this evidence, the specific contribution of environmental acoustic noise—as opposed to psychosocial or pharmacological stressors—to the disruption of complex decision-making remains insufficiently studied. Syndicus et al. (2016) found context-dependent effects on risk behavior using acoustic stressors at 60 dB(A), and Baumgart et al. (2024) demonstrated that surprising, task-irrelevant ambient sounds systematically increase risk-taking through sensory prediction mechanisms operating independently of conscious intention. However, no study to date has simultaneously examined both decision-making domains using ecologically representative high-intensity noise while incorporating objective physiological stress indicators. The present study addresses this gap.

The objective of this study was to examine the effect of experimental exposure to high-intensity urban noise (85–95 dB[A]) on performance in risk-based and intertemporal decision-making tasks in healthy adults under controlled laboratory conditions. Additionally, the study aimed to characterize the stress response through measures of perceived stress and heart rate, and to explore the relationship between physiological stress indicators and decision-making outcomes.

Based on the theoretical and empirical framework presented, three hypotheses were formulated. First, it was hypothesized that participants exposed to high-intensity urban noise would exhibit worse performance on decision-making tasks, characterized by more disadvantageous and risky choices as well as greater delay discounting, compared to participants in the silence control condition. Second, it was hypothesized that noise-exposed participants would report higher levels of perceived stress and exhibit elevated heart rate compared to control group participants, reflecting activation of the autonomic stress response. Third, it was hypothesized that elevated heart rate during the noise experimental condition would function as an objective physiological indicator of noise-induced stress and would correlate negatively with adaptive decision-making performance—such that greater cardiac activation would predict worse decision outcomes in both the risk-based and intertemporal domains.

## 2 Methodology

### 2.1 Design

The present study employed a cross-sectional, quantitative approach with a quasi-experimental between-groups design. Formally recruited participants were randomly assigned via simple randomization to one of two conditions: experimental (noise exposure) or control (silence), with gender balance maintained across groups. The control group was supplemented with participants from a pilot test of the protocol who completed the evaluation under conditions functionally equivalent to the silence condition. This between-subjects design allows systematic manipulation of the independent variable while holding all other evaluation conditions constant, and is complemented by correlational analyses examining the association between physiological activation indicators and decision-making task performance, providing an integrated neuropsychophysiological perspective consistent with the study objectives.

### 2.2 Participants

**Figure 1:**
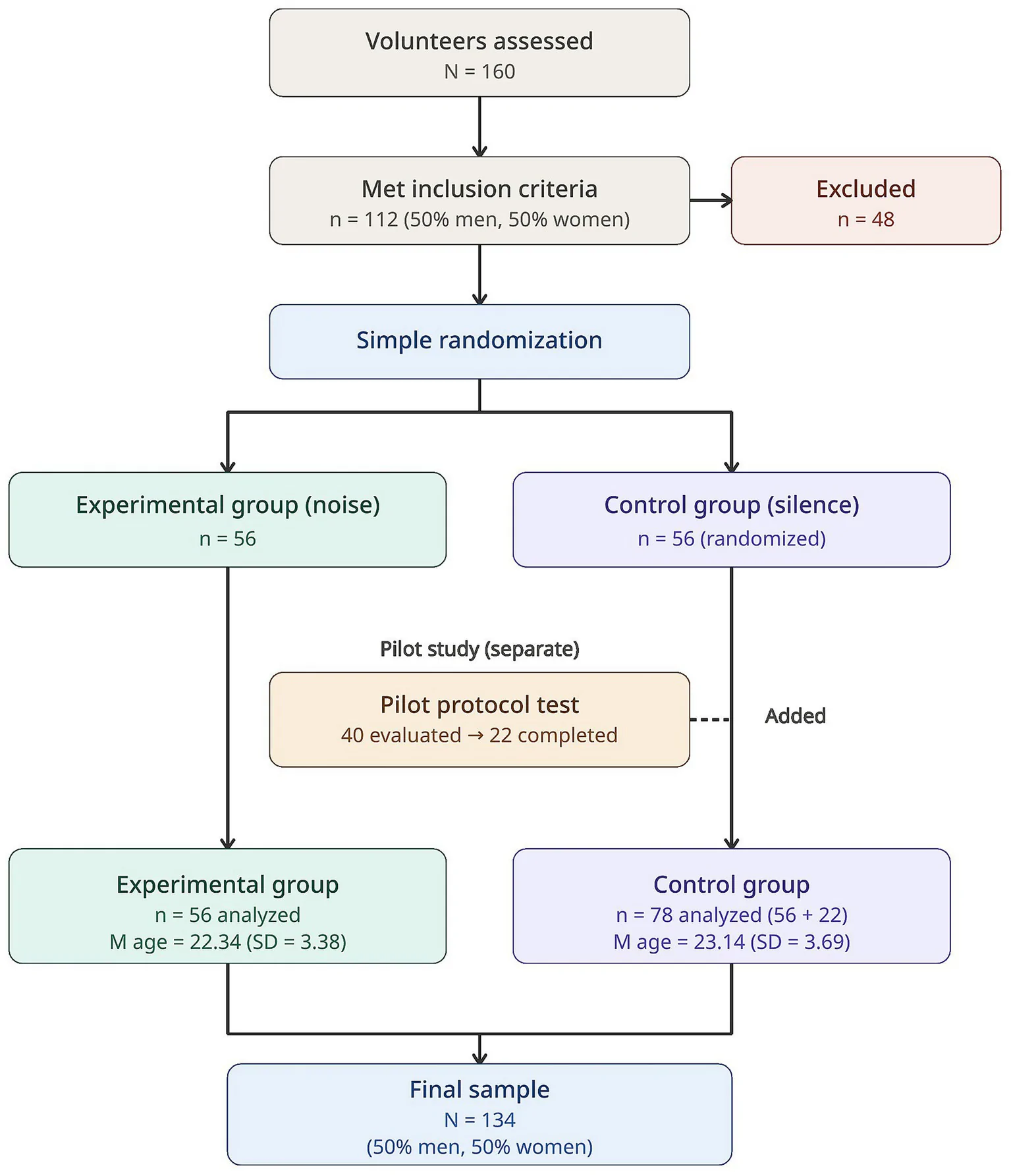
Participant flow diagram. The figure illustrates the multi-stage recruitment, screening, and allocation process. Pilot study participants (*n* = 22) were incorporated into the Control Group following successful completion of the experimental protocol under silence conditions, without randomization. *N* = total sample size; *n* = subgroup sample size; *M* = mean age in years; *SD* = standard deviation.

An *a priori* statistical power analysis conducted with G*Power (version 3.1) indicated an optimal sample size of 80 participants for the effect sizes expected in this research domain; the final sample exceeded this estimate following the multi-stage recruitment and screening process described below. A total of 160 volunteers were initially recruited, of whom 112 met the inclusion and exclusion criteria and fulfilled the gender balance requirement (50% men and 50% women). These 112 participants were randomly assigned using simple randomization to one of two experimental conditions, with gender balance maintained across groups, resulting in 56 participants per condition. Additionally, a pilot test of the experimental protocol was conducted without noise exposure with 40 participants, of whom 22 completed the protocol correctly and without procedural errors under all required conditions; these participants were incorporated into the Control Group given the functional equivalence of their evaluation conditions. The final sample thus consisted of N = 134 adult university students between 18 and 30 years of age: Experimental Group (Noise; *n* = 56, *M* age = 22.34 years, *SD* = 3.375) and Control Group (Silence; *n* = 78, *M* age = 23.14 years, *SD* = 3.693). Participants were selected through non-probabilistic convenience sampling with voluntary participation, a procedure widely used in experimental research with delimited-access populations (see Figure 1).

Exclusion criteria included the presence of medical or psychiatric conditions, sleep difficulties the night before the evaluation session, or a fasting period exceeding 3 hours prior to the session. Each evaluation session was supervised by two trained research assistants who followed a standardized protocol to ensure equivalent conditions across sessions and groups.

The groups did not differ significantly in age (U, *Z* = −1.066, *p* = 0.286), gender distribution (*χ*^2^ [1] = 0.000, *p* = 1.000), or years of schooling (Experimental: *M* = 13.02, *SD* = 2.178; Control: *M* = 14.21, *SD* = 2.880; U, *Z* = −1.368, *p* = 0.187). In the Colombian educational system, compulsory schooling covers 11 years; the remaining years correspond to university or technical education. Additionally, the groups did not differ in baseline heart rate (U, *Z* = −1.243, *p* = 0.214) or in baseline perceived stress as measured by the PSS-14 (*t*[132] = 1.163, *p* = 0.247), confirming the initial physiological and psychological equivalence between conditions prior to the experimental manipulation. The demographic and baseline equivalence between groups supports the internal validity of the experimental design.

To verify that the inclusion of pilot study participants (*n* = 22) in the Control Group did not introduce systematic bias, a sensitivity analysis was conducted excluding these participants (resulting in *n* = 56 per group). The pattern of results remained unchanged: no significant between-group differences were found for IGT Total Score (*Z* = −0.802, *p* = 0.423, *r* = 0.076) or log(*k*) MCQ (*Z* = −0.940, *p* = 0.347, *r* = 0.089), while the Experimental Group continued to show a significantly greater ΔHR than the Control Group (*t*[110] = −13.459, *p* < 0.001, *d* = 2.54), confirming that the composition of the Control Group did not affect the study’s primary conclusions.

### 2.3 Experimental context and conditions

Evaluations were conducted in two adjacent, acoustically controlled rooms: a resting room and an experimental room. The resting room was used for participant reception, informed consent signing, protocol familiarization, PSS-14 administration, and heart rate recording during the baseline and recovery phases. The experimental room was the setting in which the cognitive and behavioral evaluation instruments were administered. As a protocol control measure, an automated digital sphygmomanometer was used to record blood pressure at the beginning and end of each session (see Supplementary Material).

In the experimental condition, a looped urban ambient noise audio was played in the evaluation room, composed of ecologically representative sounds from a high-activity street: heavy vehicular traffic, horns, voices, street vendors, and construction noise. The audio was calibrated and reproduced at an intensity of 85–95 dB(A), a level corresponding to the acoustic exposure characteristic of high-density urban environments and exceeding the 53–65 dB threshold established by the World Health Organization as the limit for observable health effects (Münzel et al., 2023, 2025; World Health Organization, 2018). In the control condition, the experimental room was maintained in silence, with the same equipment and physical arrangement as in the experimental condition. All other parameters—lighting, temperature (21 °C ± 1 °C), furniture arrangement, and presence of research assistants—remained constant across conditions.

**Figure 2:**
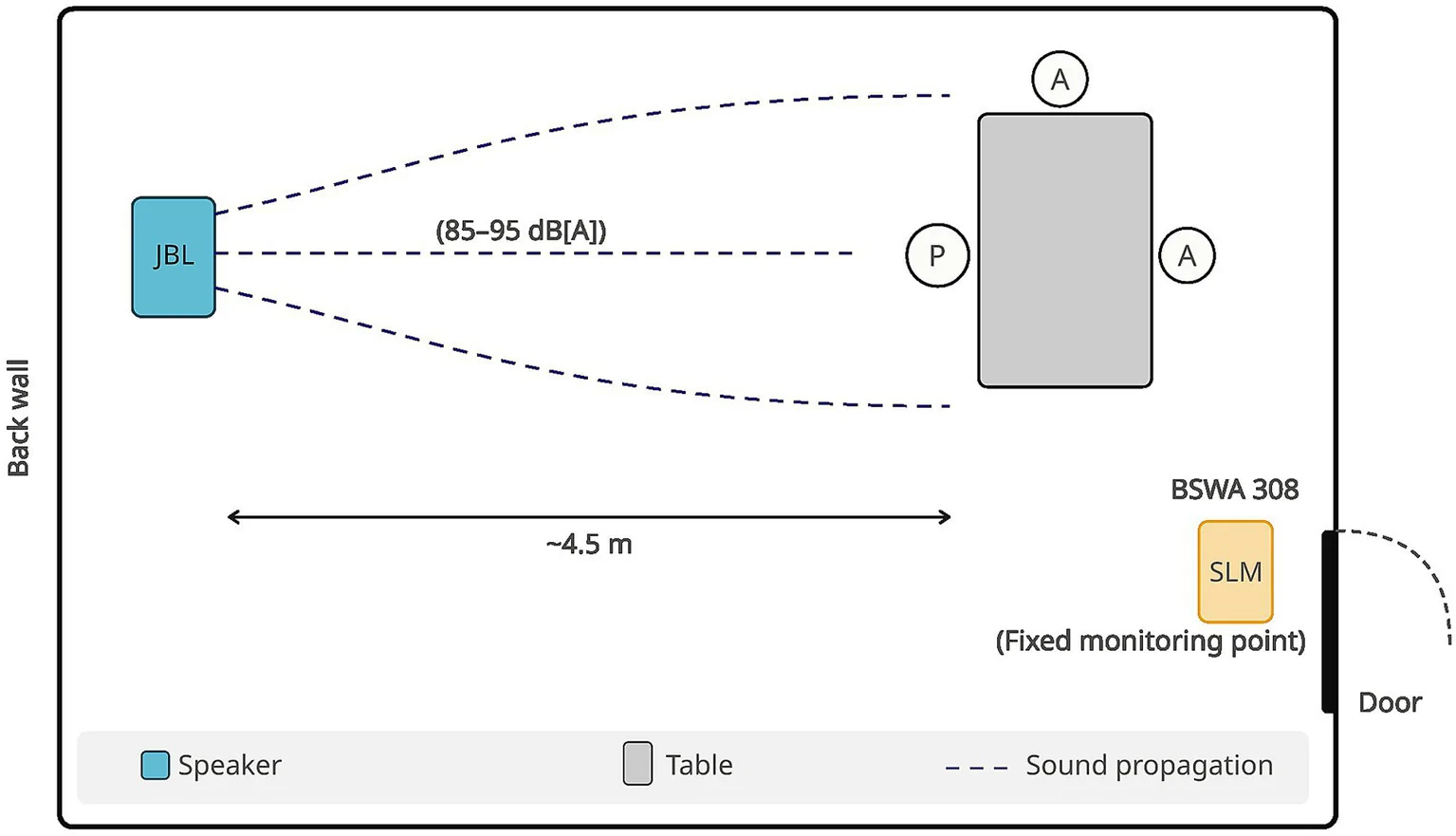
Schematic layout of the experimental room (top view). P = participant; A = research assistant; SLM = sound level meter (BSWA 308, Class 1, IEC 61672). The JBL EON ONE COMPACT loudspeaker was positioned against the back wall, approximately 4.5 m from the participant. The participant was seated with their back to the speaker. Sound pressure levels were monitored at a fixed point near the door (opposite wall) to verify maintenance of 85–95 dB(A) throughout the session.

The acoustic stimulus consisted of a 65-min loop recording of authentic urban ambient noise captured in a real high-activity street in Medellín, Colombia, comprising the full acoustic complexity of a typical Latin American city center—including heavy vehicular traffic, horns, construction noise, street vendors, and pedestrian voices—and professionally edited for experimental use. The stimulus was reproduced through a JBL EON ONE COMPACT loudspeaker (JBL Professional, USA). Sound intensity was calibrated prior to each session using a Class 1 sound level meter (BSWA 308, BSWA Technology Co., China; compliant with IEC 61672 standards) and verified at 15-min intervals throughout all experimental sessions to ensure acoustic consistency (see Figure 2). The recording incorporated the natural temporal variability characteristics of real urban soundscapes, with sound pressure levels ranging from 85 to 95 dB(A)—reflecting fluctuations between peak traffic events and brief attenuations inherent to the original recording—rather than a constant intensity signal. This variability enhances the ecological validity of the stimulus (Barrigón et al., 2018; Tiwari et al., 2024). Total exposure duration in the experimental room was approximately 45 min, consistent with laboratory stress induction protocols (Münzel et al., 2018), and does not represent a risk of permanent auditory damage according to established occupational health standards (World Health Organization, 2018).

### 2.4 Instruments

Five instruments were used to assess the variables of interest: four psychological measures and one continuous psychophysiological measure.

#### 2.4.1 Iowa gambling task

Developed by Bechara et al. (1994), the IGT is the most internationally validated instrument for assessing risk-based decision-making under uncertainty, with robust evidence of sensitivity to ventromedial prefrontal cortex function and somatic marker processing (Bechara and Damasio, 2005). Participants select 100 cards from four decks (A–D): decks A and B yield high immediate gains but net cumulative losses (disadvantageous), while decks C and D yield lower immediate gains but a net positive outcome (advantageous). Performance is indexed by the net score [(C + D) − (A + B)] across five blocks of 20 trials. Monetary values were adapted from USD to COP maintaining all structural properties of the task unchanged.

#### 2.4.2 Monetary choice questionnaire

Developed by Kirby et al. (1999), the MCQ assesses intertemporal decision-making through delay discounting. Across 27 items at three reward magnitude levels, participants choose between smaller immediate and larger delayed monetary rewards. The hyperbolic discounting *parameter k* is derived from responses by identifying the *k* value of the hyperbolic function V = A/(1 + kD) that best fits each participant’s pattern of choices, where V is the subjective value of the delayed reward, A is its objective amount, and D is the delay in days. Because the distribution of *k* is typically highly positively skewed, values were log-transformed for all analyses—yielding log(*k*)—which normalizes the distribution and stabilizes variance, as is standard practice in delay discounting research (Kirby et al., 1999; Myerson et al., 2001). Higher log(*k*) values indicate steeper discounting and greater preference for immediate rewards; one-year test–retest reliability is *r* = 0.71 in university samples (Kirby, 2009). Monetary values were adapted from USD to COP based on the exchange rate average for the month prior to administration, applied equivalently to both the MCQ and IGT.

#### 2.4.3 Perceived stress scale

Developed by Cohen et al. (1983) and adapted to Spanish by Remor (2006), the PSS-14 is a self-report scale assessing the degree to which respondents perceive their life situations as stressful, with strong psychometric properties (Cronbach’s *α* = 0.81; Remor, 2006). It was administered as a baseline measure of perceived stress prior to the experimental session. Additionally, a visual analogue scale of mood self-perception (Ríos-Flórez et al., 2019, 2021; Cronbach’s *α* = 0.87; test–retest reliability *p* = 0.05) was administered before and after task execution, on which participants rated their experienced stress on a 0–10 scale.

#### 2.4.4 Heart rate

HR was recorded using five finger pulse oximeters (Home Life FS10D, Homelife, Colombia), non-invasive medical devices measuring HR and arterial oxygen saturation (SpO₂) via optical photoplethysmography, validated for experimental estimation of HR and its temporal variations in controlled settings (Schafer and Vagedes, 2013). All devices were calibrated prior to the study. HR was recorded at six time points (1 min each): baseline rest, adaptation, task onset, mid-task, task-end, and recovery. Mean HR at each phase was calculated as the average beats recorded during the corresponding one-minute window. This multi-phase recording scheme allows tracing the participant’s autonomic activation curve across the protocol and evaluating both the magnitude of the stress response and the speed of autonomic recovery following stressor exposure. In the present study, HR serves as an indicator of sympathetic activation associated with noise-induced stress, and its variations across phases and between groups are analyzed to characterize the autonomic response to the stressor.

### 2.5 Procedure

Data collection took place between February and March 2026 at the Psychology Laboratory, Department of Psychology, Politécnico Grancolombiano University Institution, Medellín, Colombia.

The experimental protocol comprised five sequential phases: (1) reception and informed consent, including baseline blood pressure and heart rate recording; (2) pre-test administration of the PSS-14 and visual analogue stress scale; (3) experimental tasks and physiological recording, during which participants completed the MCQ followed by the IGT under noise or silence conditions, with heart rate recorded at task onset, mid-task, and task-end; (4) post-test stress assessment and autonomic recovery recording; and (5) debriefing and protocol closure. Full procedural details are provided in Supplementary Material.

### 2.6 Statistical analysis

Statistical analyses were organized to address the three study hypotheses: (H1) between-group differences in decision-making performance; (H2) between-group differences in physiological and subjective stress indicators; and (H3) associations between cardiac activation and decision-making performance, including mediation. All statistical analyses were performed using IBM SPSS Statistics (version 26.0). Prior to inferential analyses, the normality of score distributions was assessed for each group separately using the Shapiro–Wilk test and the Kolmogorov–Smirnov test with Lilliefors correction. Three variables met the normality assumption: heart rate variation (ΔHR), the Perceived Stress Scale (PSS-14), and Deck A choices on the IGT. All remaining variables violated normality and were analyzed using non-parametric procedures. The significance level was set at *α* = 0.05 for all tests. Effect sizes were calculated using Cohen’s *d* for parametric comparisons and *r* = |*Z*|/√*N* for non-parametric comparisons and interpreted according to Cohen's (1988) benchmarks: *d* or *r* ≈ 0.20/0.10 = small, 0.50/0.30 = medium, 0.80/0.50 = large.

Between-group comparisons (Experimental vs. Control) were conducted using the independent samples t-test for normally distributed variables (ΔHR, PSS-14, and Deck A choices) and the Mann–Whitney U test for the remaining variables. These comparisons were performed on all heart rate indicators across the six recording time points, stress measures (PSS-14, pre- and post-task subjective stress, and stress change), IGT performance variables (total score, individual deck choices, block net scores, and Learning Index), and the MCQ delay discounting parameter log(*k*) and consistency index.

To examine the trajectory of decisional learning across IGT trials, a repeated-measures ANOVA was conducted with net score as the dependent variable, block (1–5) as the within-subjects factor, and experimental condition as the between-subjects factor. Similarly, a repeated-measures ANOVA was conducted for heart rate, with recording time point (baseline, adaptation, task onset, mid-task, task-end, and recovery) as the within-subjects factor and experimental condition as the between-subjects factor. In both analyses, Mauchly’s test was used to evaluate the sphericity assumption; when violated, degrees of freedom were corrected using the Greenhouse–Geisser procedure. Post-hoc pairwise comparisons were conducted with Bonferroni adjustment. Multivariate test statistics (Wilks’ *Λ*) were reported as a complementary check of univariate findings.

Bivariate correlations were computed to examine the associations between physiological activation, perceived stress, and decision-making performance, organized into three variable sets. Set 1 examined correlations between HR indicators (ΔHR and Mid-task HR) and decision-making performance (IGT Total Score, Block 1 net score, Learning Index, and log[k] MCQ). Set 2 examined correlations between perceived stress measures (PSS-14 and Stress change) and decision-making performance (IGT Total Score and log[k] MCQ). Set 3 examined correlations between physiological activation and perceived stress (ΔHR and Mid-task HR with PSS-14 and Stress change). All correlations were computed using Spearman’s *ρ*, except for the ΔHR × PSS-14 pair in Set 3, for which Pearson’s *r* was used given that both variables met the normality assumption. Correlations were computed for the total sample and for each group separately.

To examine whether between-group differences in decision-making performance persisted after controlling for individual differences in baseline physiological activation and perceived stress, two analyses of covariance (ANCOVA) were conducted with IGT Total Score and log(k) MCQ as dependent variables, experimental condition as the fixed factor, and Baseline HR and PSS-14 as covariates. Homogeneity of error variances was verified using Levene’s test prior to interpretation.

Finally, two simple mediation analyses were conducted to examine whether ΔHR functioned as a mediating mechanism between experimental condition (noise vs. silence) and decision-making performance. In a simple mediation model, the independent variable is hypothesized to influence the dependent variable both directly and indirectly through a third variable—the mediator. Here, ΔHR served as the mediator, with IGT Total Score (Mediation Model 1) and log(k) MCQ (Mediation Model 2) as dependent variables. Both models were estimated using the PROCESS macro for SPSS (Model 4; Hayes, 2022), which implements ordinary least squares path analysis with bias-corrected bootstrapping (5,000 resamples) to estimate indirect effects and their 95% confidence intervals (CI). Mediation was inferred when the 95% CI for the indirect effect excluded zero.

### 2.7 Ethical considerations

The study was conducted in compliance with the Declaration of Helsinki (World Medical Association, 2013), Resolution 8430 of 1993 of the Colombian Ministry of Health—classifying the study as minimal risk research—and Law 1090 of 2006 regulating the practice of Psychology in Colombia. Voluntary participation, the right to withdraw without consequences, data confidentiality through anonymization, and the exclusive use of information for scientific purposes were guaranteed. All participants provided written informed consent. The sound intensity employed (85–95 dB) over a maximum of 30 min does not represent a risk of permanent auditory damage according to available evidence. A complete debriefing was conducted at the end of each session.

## 3 Results

Between-group comparisons, repeated-measures analyses of variance (RM-ANOVA), bivariate correlations, analyses of covariance (ANCOVA), and bootstrapped mediation analyses were conducted to examine the effect of experimental condition on physiological, subjective, and decision-making performance variables. Distributional normality was assessed for each group separately using the Shapiro–Wilk test and the Kolmogorov–Smirnov test with Lilliefors correction. Three variables met the normality assumption: ΔHR, PSS-14, and Deck A choices; all remaining variables were analyzed using non-parametric procedures. The significance level was set at *α* = 0.05 for all tests. Effect sizes were calculated using Cohen’s *d* for parametric comparisons and *r* = |*Z*|/√*N* for non-parametric comparisons, interpreted according to Cohen's (1988) benchmarks: *d* or *r* ≈ 0.20/0.10 = small, 0.50/0.30 = medium, 0.80/0.50 = large.

### 3.1 IGT learning trajectory: repeated-measures

**Figure 3:**
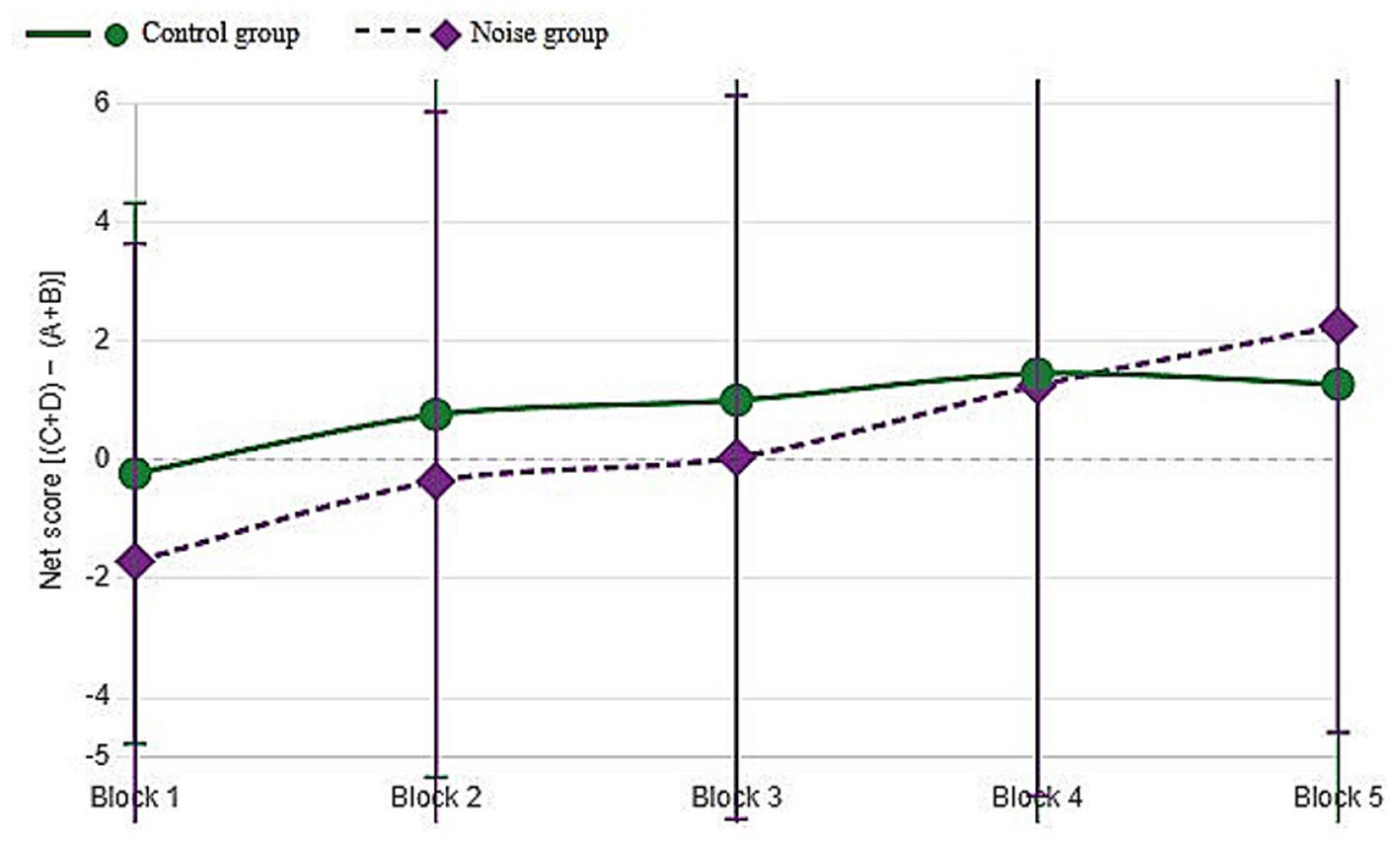
IGT net score by block and experimental condition. Mean net score [(C + D) − (A + B)] across five blocks of 20 trials each. Positive values indicate advantageous choices (Decks C and D); negative values indicate disadvantageous choices (Decks A and B). Error bars represent ±1 SD. The horizontal dashed reference line at zero marks the threshold between advantageous and disadvantageous performance.

To examine whether the decisional learning trajectory across IGT trials differed between groups, a repeated-measures ANOVA was conducted with net score as the dependent variable, trial block (1–5) as the within-subjects factor, and experimental condition as the between-subjects factor. Descriptive statistics by block and group are presented in Table 1, ANOVA results in Table 2, and the net score profile across blocks is illustrated in Figure 3. Given that Mauchly’s test was significant (*W* = 0.755, *p* < 0.001), the Greenhouse–Geisser correction was applied (*ε* = 0.932). Results revealed a significant main effect of Block, *F*(3.589, 473.786) = 3.591, *p* = 0.009, *η*^2^*p* = 0.026, indicating that IGT net score varied across the five blocks regardless of experimental condition, confirmed by multivariate tests (Wilks’ *Λ* = 0.906, *F*[4, 129] = 3.366, *p* = 0.012, *η*^2^*p* = 0.094). This pattern reflects a progressive learning effect: both groups tended to increase advantageous choices over the course of trials, as shown in Figure 3. No significant main effect of experimental condition was observed, *F*(1, 132) = 0.989, *p* = 0.322, *η*^2^*p* = 0.007, nor a significant Block × Group interaction, *F*(3.589, 473.786) = 0.757, *p* = 0.541, *η*^2^*p* = 0.006, confirmed by multivariate tests (Wilks’ *Λ* = 0.982, *F*[4, 129] = 0.597, *p* = 0.666, *η*^2^*p* = 0.018). Nevertheless, the descriptive analysis reveals a theoretically interesting pattern: the Noise Group started with a lower net score in Block 1 (*M* = −1.71, *SD* = 5.345) compared to the Control Group (*M* = −0.23, *SD* = 4.547), while in Block 5 the Noise Group reached a higher score (*M* = 2.25, *SD* = 6.834 vs. *M* = 1.27, *SD* = 7.595), suggesting a possible initial disadvantage progressively compensated through feedback-based learning.

**Table 1:** Descriptive statistics for IGT net score by block and experimental condition.

Block	Experimental group		Control group	
	M	SD	M	SD
Block 1 (trials 1–20)	−1.71	5.345	−0.23	4.547
Block 2 (trials 21–40)	−0.36	6.210	0.76	6.099
Block 3 (trials 41–60)	0.04	6.084	1.00	7.329
Block 4 (trials 61–80)	1.25	6.903	1.45	7.676
Block 5 (trials 81–100)	2.25	6.834	1.27	7.595

M: mean net score [(C + D) − (A + B)] per block; SD: standard deviation. Positive values indicate a predominance of advantageous choices (Decks C and D); negative values indicate disadvantageous choices (Decks A and B).

**Table 2:** Results of the repeated-measures ANOVA for IGT net score.

Source	*F*	*df*	*p*	*η* ^2^ *p*
Univariate (Greenhouse–Geisser, *ε* = 0.932)				
Block	3.591	3.589, 473.786	0.009**	0.026
Group	0.989	1, 132	0.322	0.007
Block × Group	0.757	3.589, 473.786	0.541	0.006
Multivariate				
Block (Wilks’ *Λ*)	3.366	4, 129	0.012	0.094
Block × Group (Wilks’ *Λ*)	0.597	4, 129	0.666	0.018

*F* = Fisher’s *F*-ratio statistic; df: degrees of freedom; *η*^2^*p* = partial eta squared. Univariate degrees of freedom for Block and Block × Group were corrected using Greenhouse–Geisser (*ε* = 0.932) due to violation of the sphericity assumption (Mauchly’s *W* = 0.755, *p* < 0.001). ES interpretation: *η*^2^*p* ≈ 0.01 = small, 0.06 = medium, 0.14 = large (Cohen, 1988); *p ≤ 0.05; **p ≤ 0.01; ***p ≤ 0.001.

### 3.2 Heart rate response: repeated-measures

**Figure 4:**
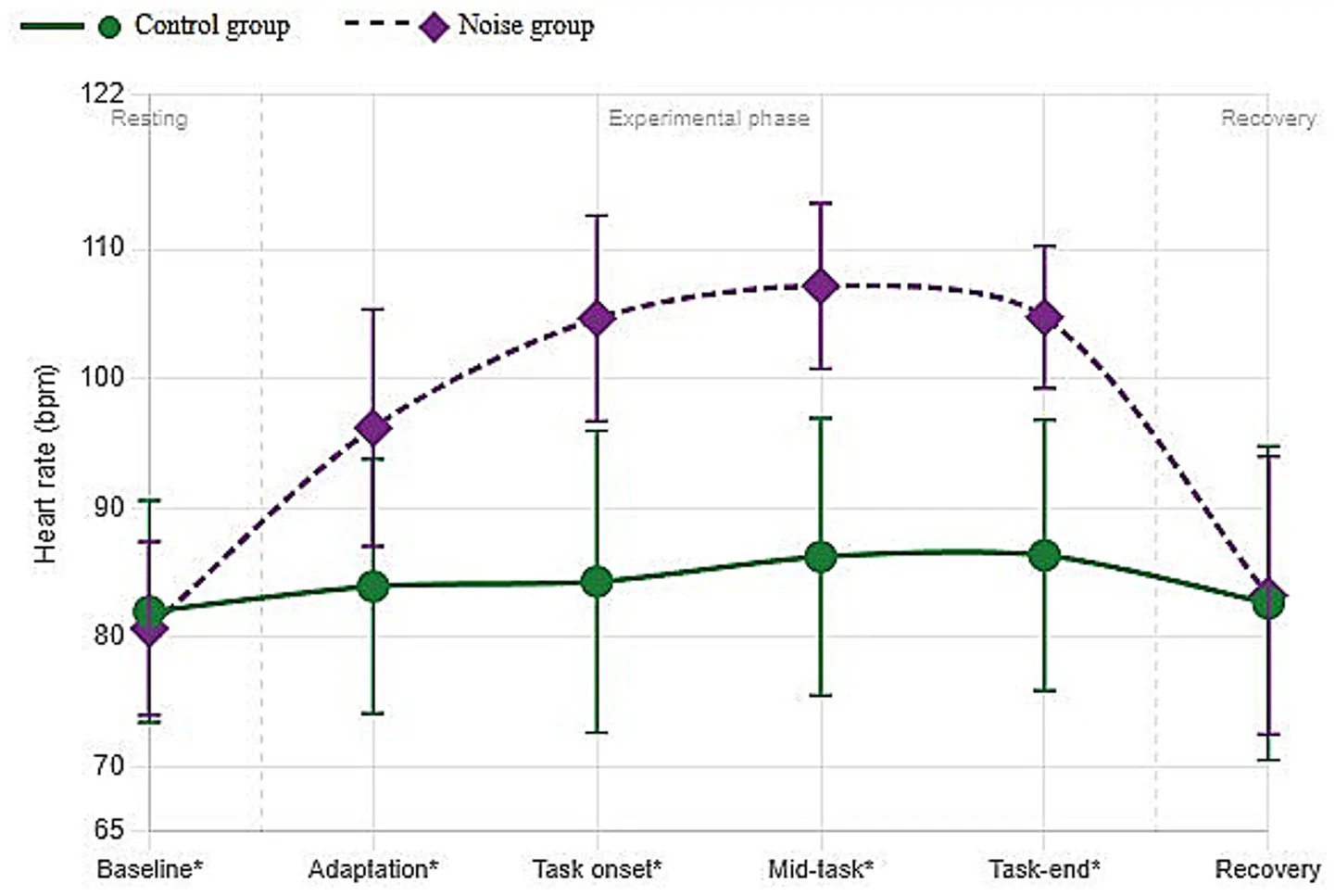
Heart rate profile by recording time point and experimental condition. Estimated marginal means (bpm) at each phase of the experimental protocol; bpm = beats per minute. Vertical dashed lines delimit the three phases: resting phase (left), experimental phase (centre), and recovery phase (right). Asterisks (*) indicate significant between-group differences at each time point (*p <* 0.001, Bonferroni adjustment). Error bars represent ±1 SD.

The autonomic response across the experimental protocol was examined using a repeated-measures ANOVA with HR as the dependent variable, recording time point as the within-subjects factor, and experimental condition as the between-subjects factor. Descriptive statistics are presented in Table 3, ANOVA results in Table 4, post-hoc comparisons in Table 5, and the HR profile across the protocol is illustrated in Figure 4. Given that Mauchly’s test was significant (W = 0.281, *p* < 0.001), the Greenhouse–Geisser correction was applied (*ε* = 0.660). Results revealed significant effects for all three terms of the model. The main effect of Time was significant, *F*(3.186, 420.584) = 117.957, *p* < 0.001, *η*^2^*p* = 0.472. The main effect of Group was also significant, *F*(1, 132) = 89.000, *p* < 0.001, *η*^2^*p* = 0.403. The Time × Group interaction was significant, *F*(3.186, 420.584) = 66.649, *p* < 0.001, *η*^2^*p* = 0.336, indicating that HR trajectories differed significantly between conditions. These results were confirmed by multivariate tests (Wilks’ *Λ* = 0.185, *F*[5, 128] = 112.474, *p* < 0.001, *η*^2^*p* = 0.815). Post-hoc comparisons with Bonferroni adjustment (Table 5) showed that HR during all time points of the experimental phase was significantly higher than at baseline and recovery (all *p* < 0.001), while recovery HR did not differ significantly from baseline (*p* = 0.641), evidencing complete autonomic recovery. As shown in Figure 4, the Experimental Group showed a progressive HR elevation from the adaptation phase (*M* = 96.20, *SD* = 9.160), reaching its maximum at Mid-task HR (*M* = 107.16, *SD* = 6.387), with differences relative to the Control Group ranging between 12 and 21 bpm during the experimental phase, while the Control Group maintained stable HR (range: 82.01–86.35 bpm).

**Table 3:** Descriptive statistics for heart rate by recording time point and experimental condition.

Time point	Experimental group		Control group	
	M	SD	M	SD
Baseline HR	80.70	6.696	82.01	8.568
Adaptation HR	96.20	9.160	83.95	9.830
Task onset HR	104.66	7.941	84.32	11.661
Mid-task HR	107.16	6.387	86.24	10.716
Task-end HR	104.75	5.488	86.35	10.454
Recovery HR	83.25	10.740	82.65	12.131

M: mean heart rate (HR) in beats per minute (bpm); SD: standard deviation.

**Table 4:** Results of the repeated-measures ANOVA for heart rate.

Source	*F*	*df*	*p*	*η* ^2^ *p*
Univariate (Greenhouse–Geisser, *ε* = 0.660)				
Time	117.957***	3.186, 420.584	<0.001	0.472
Group	89.000***	1, 132	<0.001	0.403
Time × Group	66.649***	3.186, 420.584	<0.001	0.336
Multivariate				
Time (Wilks’ *Λ*)	112.474***	5, 128	<0.001	0.815

*F* = Fisher’s *F*-ratio statistic; df: degrees of freedom; *η*^2^*p*: partial eta squared. Univariate degrees of freedom for Time and Time × Group were corrected using Greenhouse–Geisser (*ε* = 0.660) due to violation of the sphericity assumption (Mauchly’s *W* = 0.281, *p* < 0.001). Multivariate results confirmed univariate findings (Wilks’ *Λ* = 0.185). ES interpretation: *η*^2^*p* ≈ 0.01 = small, 0.06 = medium, 0.14 = large (Cohen, 1988); *p ≤ 0.05; **p ≤ 0.01; ***p ≤ 0.001.

**Table 5:** Pairwise comparisons (Bonferroni) between heart rate recording time points.

Comparison	Mean difference	SE	*p*
Baseline vs. Adaptation	−8.718***	0.572	<0.001
Baseline vs. Task onset	−13.136***	0.871	<0.001
Baseline vs. Mid-task	−15.348***	0.783	<0.001
Baseline vs. Task-end	−14.193***	0.757	<0.001
Baseline vs. Recovery	−1.597	0.781	0.641
Adaptation vs. Task onset	−4.418***	0.953	<0.001
Adaptation vs. Mid-task	−6.630***	0.903	<0.001
Adaptation vs. Task-end	−5.476***	0.882	<0.001
Adaptation vs. Recovery	7.121***	0.890	<0.001
Task onset vs. Mid-task	−2.212*	0.693	0.026
Task onset vs. Task-end	−1.057	0.758	1.000
Task onset vs. Recovery	11.539***	1.184	<0.001
Mid-task vs. Task-end	1.154	0.564	0.642
Mid-task vs. Recovery	13.750***	1.034	<0.001
Task-end vs. Recovery	12.596***	1.084	<0.001

SE: standard error. Bonferroni adjustment applied for multiple comparisons. Mean differences expressed in beats per minute (bpm); *p* = 1.000 indicates complete absence of significant difference; *p ≤ 0.05; **p ≤ 0.01; ***p ≤ 0.001.

### 3.3 Between-group comparisons: heart rate and stress variables

To evaluate whether the noise manipulation effectively induced a stress response, between-group differences were examined across both physiological and subjective stress indicators. Noise exposure was expected to produce measurable increases in heart rate and perceived stress relative to the silence condition, as evidence of autonomic and psychological activation. Between-group comparisons on heart rate and stress variables are presented in Table 6. The groups did not differ significantly in Baseline HR (*Z* = −1.243, *p* = 0.214, *r* = 0.107), confirming initial physiological equivalence between conditions. From the adaptation phase onward, the Experimental Group exhibited significantly higher HR at all task time points: Adaptation HR (*Z* = −6.355, *p* < 0.001, *r* = 0.549), Task onset HR (*Z* = −8.992, *p* < 0.001, *r* = 0.777), Mid-task HR (*Z* = −9.661, *p* < 0.001, *r* = 0.835), and Task-end HR (*Z* = −9.500, *p* < 0.001, *r* = 0.821), with large to very large effect sizes throughout. ΔHR was significantly greater in the Experimental Group (*M* = 26.48, *SD* = 9.097) than in the Control Group (*M* = 4.23, *SD* = 8.762), *t*(132) = 14.269, *p* < 0.001, *d* = 2.50, 95% *CI* [19.167, 25.336], constituting the largest effect observed in the study. During recovery, groups did not differ in HR (*Z* = −0.088, *p* = 0.930, *r* = 0.008), evidencing complete autonomic recovery. Regarding perceived stress, baseline PSS-14 scores did not differ between groups (*t*[132] = 1.163, *p* = 0.247, *d* = 0.20). However, Pre-task subjective stress (*Z* = −4.294, *p* < 0.001, *r* = 0.371), Post-task stress perception (*Z* = −9.911, *p* < 0.001, *r* = 0.856), and Stress change post−pre (*Z* = −9.301, *p* < 0.001, *r* = 0.803) were all significantly higher in the Experimental Group, with medium to very large effect sizes.

**Table 6:** Between-group comparisons on heart rate and stress variables.

Variable	Experimental group		Control group		Statistic	*p*	ES
	M	SD	M	SD			
Baseline HR	80.70	6.696	82.01	8.568	*Z*(U) = −1.243	0.214	*r* = 0.107
Adaptation HR	96.20	9.160	83.95	9.830	*Z*(U) = −6.355***	<0.001	*r* = 0.549
Task onset HR	104.66	7.941	84.32	11.661	*Z*(U) = −8.992***	<0.001	*r* = 0.777
Mid-task HR	107.16	6.387	86.24	10.716	*Z*(U) = −9.661***	<0.001	*r* = 0.835
Task-end HR	104.75	5.488	86.35	10.454	*Z*(U) = −9.500***	<0.001	*r* = 0.821
Recovery HR	83.25	10.740	82.65	12.131	*Z*(U) = −0.088	0.930	*r* = 0.008
ΔHR (Mid − Baseline)	26.48	9.097	4.23	8.762	*t*(T) = 14.269***	<0.001	*d* = 2.50
PSS-14	19.04	11.288	16.96	9.318	*t*(T) = 1.163	0.247	*d* = 0.20
Pre-task subjective stress	1.45	1.008	0.73	0.963	*Z*(U) = −4.294***	<0.001	*r* = 0.371
Post-task stress perception	9.71	0.594	2.92	2.289	*Z*(U) = −9.911***	<0.001	*r* = 0.856
Stress change^a^	8.27	1.272	2.19	2.186	*Z*(U) = −9.301***	<0.001	*r* = 0.803

M: mean; SD: standard deviation; ES: effect size; a: Stress change (post−pre); *Z*(U): *Z* statistic from the Mann–Whitney U test; *t*(T): *t* statistic from the independent samples *t*-test; *r*: |*Z*|/√*N*; *d*: Cohen’s *d*; HR: heart rate in beats per minute (bpm); ΔHR: heart rate variation (Mid-task HR − Baseline HR); PSS-14: 14-item Perceived Stress Scale; Pre-task subjective stress and post-task stress perception were measured on a 0–10 visual analogue scale; ES interpretation for *r*: 0.10 = small, 0.30 = medium, 0.50 = large; ES interpretation for *d*: 0.20 = small, 0.50 = medium, 0.80 = large (Cohen, 1988); ^a^Stress chance -difference between post-task and pre-task scores.*p ≤ 0.05; **p ≤ 0.01; ***p ≤ 0.001.

Taken together, these findings confirm that the experimental manipulation was effective: noise exposure produced a robust, objectively measurable autonomic stress response—evidenced by large HR increases throughout the task—as well as a marked elevation in subjective stress perception, while both indicators remained stable and equivalent in the Control Group.

### 3.4 Between-group comparisons: decision-making variables

Between-group comparisons on decision-making variables are presented in Table 7. No IGT variable showed significant between-group differences. IGT Total Score did not differ between the Experimental Group (*M* = 1.46, *SD* = 14.016) and the Control Group (*M* = 4.24, *SD* = 17.207), *Z* = −0.262, *p* = 0.793, *r* = 0.023. Individual deck choices were likewise non-significant: Deck A (*t*[132] = −0.241, *p* = 0.810, *d* = 0.04), Deck B (*Z* = −1.240, *p* = 0.215, *r* = 0.107), Deck C (*Z* = −1.389, *p* = 0.165, *r* = 0.120), and Deck D (*Z* = −0.305, *p* = 0.760, *r* = 0.026). The Learning Index also showed no significant between-group differences (*Z* = −1.685, *p* = 0.092, *r* = 0.146). Notably, block-level analysis revealed a significant difference only in Block 1 (*Z* = −2.175, *p* = 0.030, *r* = 0.188), where the Experimental Group obtained a lower net score (*M* = −1.71, *SD* = 5.345) than the Control Group (*M* = −0.23, *SD* = 4.547), though with a small effect size; Blocks 2 through 5 showed no significant between-group differences (all *p* > 0.05). Regarding the MCQ, log(*k*) did not differ between groups (*Z* = −1.525, *p* = 0.127, *r* = 0.132), nor did MCQ Consistency (*Z* = −0.615, *p* = 0.538, *r* = 0.053).

**Table 7:** Between-group comparisons on decision-making variables.

Variable	Experimental group		Control group		Statistic	*p*	ES
	M	SD	M	SD			
IGT total score	1.46	14.016	4.24	17.207	*Z*(U) = −0.262	0.793	*r* = 0.023
Deck A choices	19.41	7.073	19.71	6.879	*t*(T) = −0.241	0.810	*d* = 0.04
Deck B choices	29.86	7.756	27.94	7.470	*Z*(U) = −1.240	0.215	*r* = 0.107
Deck C choices	22.70	5.319	24.36	6.798	*Z*(U) = −1.389	0.165	*r* = 0.120
Deck D choices	28.04	7.549	27.53	6.588	*Z*(U) = −0.305	0.760	*r* = 0.026
Learning Index	3.96	9.338	1.50	9.864	*Z*(U) = −1.685	0.092	*r* = 0.146
log(*k*) - MCQ	−5.072	0.338	−5.159	0.289	*Z*(U) = −1.525	0.127	*r* = 0.132
MCQ consistency	0.552	0.216	0.560	0.174	*Z*(U) = −0.615	0.538	*r* = 0.053

M: mean; SD: standard deviation; ES: effect size; *Z*(U): *Z* statistic from the Mann–Whitney U test; *t*(T): t statistic from the independent samples *t*-test; *r*: |*Z*|/√*N*; *d*: Cohen’s *d*; IGT: Iowa Gambling Task; IGT Total Score: net score [(C + D) − (A + B)] across 100 trials; Learning Index [IGT]: net score Block 5 − Block 1; log(*k*): logarithm of the hyperbolic discounting parameter from the MCQ; MCQ consistency reported on a 0–1 scale; ES interpretation for *r*: 0.10 = small, 0.30 = medium, 0.50 = large; ES interpretation for *d*: 0.20 = small, 0.50 = medium, 0.80 = large (Cohen, 1988).

### 3.5 Bivariate correlations

To examine associations between physiological activation, perceived stress, and decision-making performance, Spearman correlations were computed for variable pairs in which at least one variable violated the normality assumption, and Pearson’s *r* was used for the ΔHR × PSS-14 pair in which both variables met normality. All correlations were computed for the total sample and for each group separately (Tables 8–10).

**Table 8:** Spearman correlations between heart rate indicators and decision-making performance by group—Set 1.

Variable pair	Total sample		Experimental group		Control group	
	*ρ*	*p*	*ρ*	*p*	*ρ*	*p*
ΔHR × IGT Total Score	−0.047	0.591	0.027	0.843	−0.085	0.459
ΔHR × Block 1 net score	−0.158	0.068	−0.072	0.598	0.046	0.687
ΔHR × Learning Index	0.107	0.217	0.199	0.142	−0.160	0.163
ΔHR × log(*k*) MCQ	0.088	0.312	0.002	0.988	−0.034	0.769
Mid-task HR × IGT Total Score	−0.057	0.515	0.102	0.455	−0.168	0.141
Mid-task HR × log(*k*) MCQ	0.064	0.462	−0.044	0.748	−0.091	0.426

*ρ*: Spearman correlation coefficient; ΔHR: heart rate variation (Mid-task HR − Baseline HR); log(*k*): logarithm of the hyperbolic discounting parameter from the MCQ; Learning Index [IGT]: net score Block 5 − Block 1; All correlations were non-significant (*p* > 0.05).

**Table 9:** Spearman correlations between perceived stress and decision-making performance by group—Set 2.

Variable pair	Total sample		Experimental group		Control group	
	*ρ*	*p*	*ρ*	*p*	*ρ*	*p*
PSS-14 × IGT total score	−0.010	0.910	−0.132	0.332	0.070	0.540
PSS-14 × log(*k*) MCQ	−0.072	0.411	−0.145	0.286	−0.009	0.938
Stress change × IGT total score	0.048	0.584	0.030	0.824	0.140	0.221
Stress change × log(*k*) MCQ	0.063	0.470	−0.173	0.201	−0.065	0.575

*ρ*: Spearman correlation coefficient; PSS-14: 14-item Perceived Stress Scale; Stress change: difference between post-task and pre-task scores on the 0–10 visual analogue scale: log(*k*): logarithm of the hyperbolic discounting parameter from the MCQ; All correlations were non-significant (*p* > 0.05).

**Table 10:** Correlations between physiological activation and perceived stress by group—Set 3.

Variable pair	Total sample		Experimental group		Control group	
	Coefficient	*p*	Coefficient	*p*	Coefficient	*p*
ΔHR × PSS-14	0.079 *r*	0.367	0.008 *r*	0.955	−0.007 *r*	0.954
ΔHR × Stress change	0.637*** *ρ*	<0.001	−0.139 ρ	0.308	−0.053 *ρ*	0.648
Mid-task HR × Stress change	0.684*** *ρ*	<0.001	−0.066 ρ	0.627	0.024 *ρ*	0.838

ΔHR: heart rate variation (Mid-task HR − Baseline HR); PSS-14: 14-item Perceived Stress Scale; Stress change: difference between post-task and pre-task scores on the 0–10 visual analogue scale; Pearson (*r*) was used for ΔHR × PSS-14 as both variables met the normality assumption; Spearman (*ρ*) was used for remaining pairs; Significant correlations in the total sample reflect between-group variance and not individual-level covariation within each condition (see Results text); *p ≤ 0.05; **p ≤ 0.01; ***p ≤ 0.001.

In Set 1 (Table 8), no correlation between HR indicators (ΔHR, Mid-task HR) and decision-making performance (IGT Total Score, Block 1 net score, Learning Index, log[*k*] MCQ) reached statistical significance at any of the three levels of analysis (all *p* > 0.05). In Set 2 (Table 9), no correlation between perceived stress measures (PSS-14, Stress change post−pre) and decision-making performance (IGT Total Score, log[*k*] MCQ) was significant in either group or in the total sample (all *p* > 0.05). Taken together, these results indicate that neither physiological activation nor subjective stress predicted decision-making performance under the conditions of the present study. Set 3 (Table 10) examined associations between physiological activation and perceived stress. The Pearson correlation between ΔHR and PSS-14 was non-significant in either group or the total sample (*r* = 0.079, *p* = 0.367). However, significant positive correlations were observed between ΔHR and Stress change (*ρ* = 0.637, *p* < 0.001) and between Mid-task HR and Stress change (ρ = 0.684, *p* < 0.001) in the total sample. This pattern disappeared when groups were analyzed separately—where no correlation reached statistical significance—indicating that the correlations observed in the total sample constitute a statistical artifact produced by the marked between-group difference in both dimensions, and do not reflect individual-level covariation within each experimental condition.

### 3.6 ANCOVA

To examine whether between-group differences in decision-making performance persisted after controlling for baseline cardiac activation and pre-task perceived stress, two ANCOVAs were conducted with IGT Total Score and log(*k*) MCQ as dependent variables, experimental condition as the fixed factor, and Baseline HR and PSS-14 as covariates (Table 11). Levene’s test was non-significant for both dependent variables (IGT: *F*[1, 132] = 0.185, *p* = 0.667; log[*k*]: *F*[1, 132] = 0.873, *p* = 0.352), confirming the homogeneity of variances assumption. Results indicated that, after controlling for covariates, the effect of experimental condition did not reach statistical significance for either IGT Total Score, *F*(1, 130) = 1.212, *p* = 0.273, *η*^2^*p* = 0.009, or log(*k*) MCQ, *F*(1, 130) = 2.453, *p* = 0.120, *η*^2^*p* = 0.019. No covariate showed a significant effect on either dependent variable. Estimated marginal means were similar between groups for both IGT (Experimental Group: *M* = 1.271, *SE* = 2.149, 95% *CI* [−2.981, 5.522]; Control Group: *M* = 4.383, *SE* = 1.818, 95% *CI* [0.786, 7.980]) and MCQ (Experimental Group: *M* = −5.072, *SE* = 0.042, 95% *CI* [−5.155, −4.990]; Control Group: *M* = −5.158, *SE* = 0.035, 95% *CI* [−5.228, −5.088]), confirming the absence of significant differences in decision-making performance after controlling for individual levels of physiological activation and perceived stress.

**Table 11:** ANCOVA results for decision-making performance controlling for baseline heart rate and perceived stress.

Source	IGT total score				log(*k*) MCQ			
	*F*	*df*	*p*	*η* ^2^ *p*	*F*	*df*	*p*	*η* ^2^ *p*
Baseline HR	1.274	1, 130	0.261	0.010	0.736	1, 130	0.393	0.006
PSS-14	0.058	1, 130	0.809	0.000	0.367	1, 130	0.546	0.003
Group	1.212	1, 130	0.273	0.009	2.453	1, 130	0.120	0.019
Estimated marginal means								
Experimental group	1.271 (2.149)				−5.072 (0.042)			
Control group	4.383 (1.818)				−5.158 (0.035)			

Values in parentheses for estimated marginal means represent standard errors (SE). Covariates evaluated at their mean values: Baseline HR = 81.46 bpm; PSS-14 = 17.83. Levene’s test of error variance homogeneity was non-significant for both dependent variables: IGT Total Score *F* (1, 132) = 0.185, *p* = 0.667; log(*k*) MCQ *F*(1, 132) = 0.873, *p* = 0.352; *F* = Fisher’s *F*-ratio statistic; df: degrees of freedom; η^2^p: partial eta squared; PSS-14: 14-item Perceived Stress Scale; log(*k*): logarithm of the hyperbolic discounting parameter from the MCQ.

### 3.7 Mediation analysis

**Figure 5:**
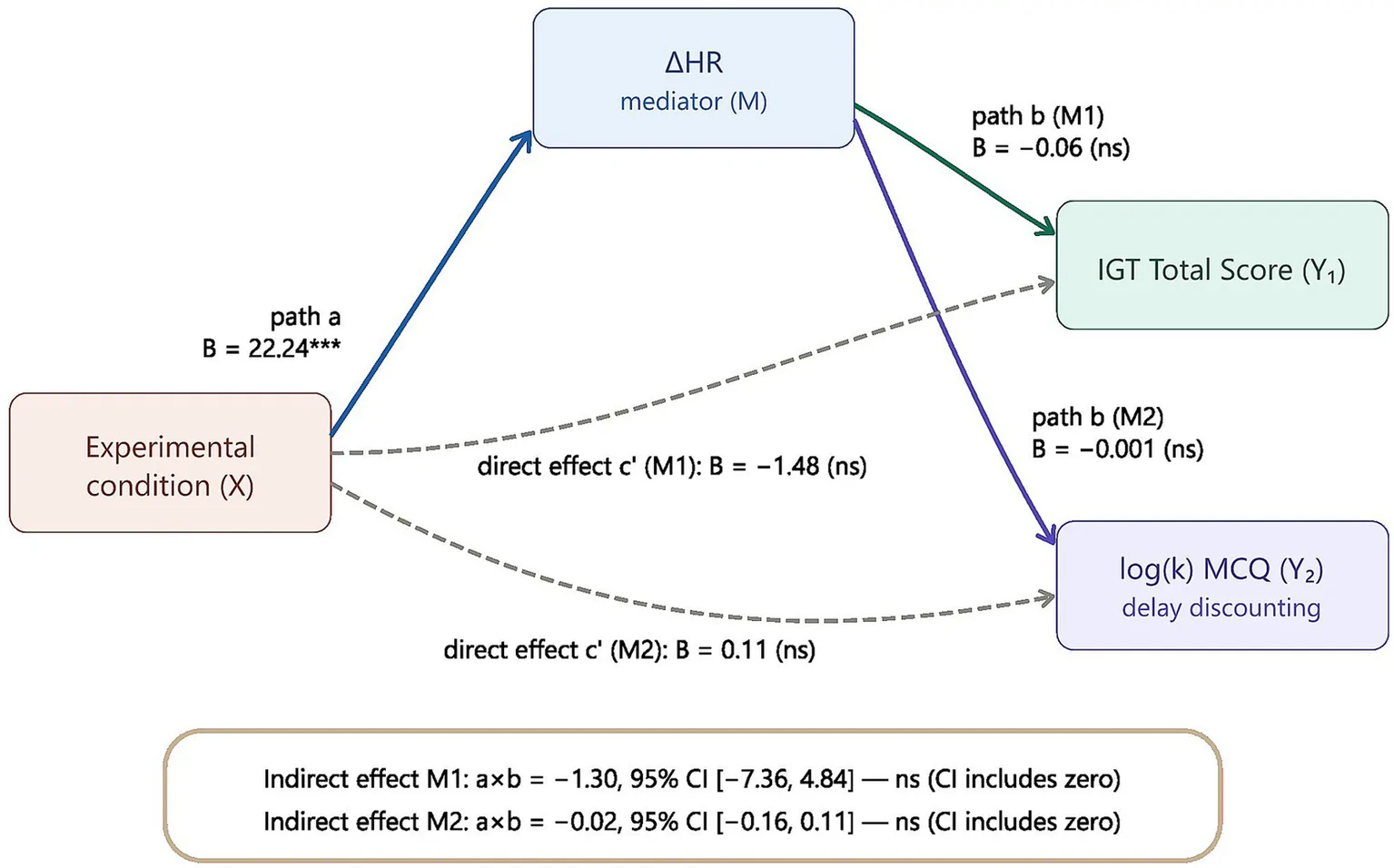
Simple mediation model. Simple mediation model (Hayes Model 4) testing ΔHR as a mediator between experimental condition and decision-making performance; *X*: experimental condition (1 = Noise, 0 = Silence); *M*: ΔHR (heart rate variation: Mid-task HR − Baseline HR, in bpm); *Y*₁: IGT Total Score; *Y*₂: log(*k*) MCQ (delay discounting parameter); Solid lines represent paths *a*, and *b*; Dashed lines represent direct effects (*c*’); Unstandardized regression coefficients (*B*) are shown for each path; Indirect effects were estimated using bias-corrected bootstrapping with 5,000 samples; 95% confidence intervals (*CI*) are reported at the bottom of the figure; ns: non-significant (95% *CI* includes zero); *** *p ≤* 0.001.

Simple mediation analyses (Model 4; Hayes, 2022) were conducted to examine whether autonomic activation induced by noise exposure—operationalized as heart rate variation (ΔHR)—mediated the relationship between experimental condition and decision-making performance. Two models were estimated using bias-corrected bootstrapping with 5,000 resamples and a fixed seed (12345) to ensure reproducibility (Table 12 and Figure 5).

**Table 12:** Simple mediation analysis (Hayes Model 4).

Path	Model 1: IGT				Model 2: log(*k*) MCQ			
	*B*	SE	*t*	*p*	*B*	SE	*t*	*p*
*a* Group → ΔHR	22.240***	1.547	14.377	<0.001	22.240***	1.547	14.377	<0.001
*b* ΔHR → Y (controlling Group)	−0.058	0.158	−0.369	0.713	−0.001	0.003	−0.340	0.735
*c*’ Direct effect Group → Y	−1.484	4.491	−0.330	0.742	0.110	0.087	1.258	0.211
*c* Total effect Group → Y	−2.779	2.795	−0.995	0.322	0.087	0.054	1.595	0.113
Indirect effect (*a* × *b*)	−1.295	3.109	–	–	−0.023	0.068	–	–
95% Bootstrap CI	[−7.356, 4.837]				[−0.158, 0.113]			

ΔHR as mediator between experimental condition and decision-making performance (*N* = 134, bootstrap = 5,000 samples); *B:* unstandardized regression coefficient; SE: standard error; *t:* Student’s t statistic. Group: 1 = Experimental Group (Noise), 0 = Control Group (Silence); ΔHR: heart rate variation (Mid-task HR − Baseline HR, in bpm); *Y* = dependent variable: IGT Total Score (Model 1) or log(*k*) MCQ (Model 2); Bootstrap confidence intervals (CI) were computed with 5,000 samples (Hayes, 2022); Mediation is considered statistically significant when the 95% CI does not include zero; –: not applicable; *p ≤ 0.05; **p ≤ 0.01; ***p ≤ 0.001.

In Model 1, with IGT Total Score as the dependent variable, path a was significant: the noise condition predicted a significant increase in ΔHR (*B* = 22.240, *SE* = 1.547, *t* = 14.377, *p* < 0.001), confirming the effect of the acoustic stressor on cardiac activation. However, path b was non-significant: ΔHR did not predict IGT Total Score when controlling for experimental condition (*B* = −0.058, *SE* = 0.158, *t* = −0.369, *p* = 0.713). The direct effect of experimental condition on IGT performance was also non-significant (*c*’ = −1.484, *SE* = 4.491, *t* = −0.330, *p* = 0.742), as was the total effect (c = −2.779, *SE* = 2.795, *t* = −0.995, *p* = 0.322). The indirect effect was −1.295 (*SE* = 3.109, 95% *CI* [−7.356, 4.837]), with the confidence interval including zero, indicating that mediation was not statistically significant.

In Model 2, with log(*k*) MCQ as the dependent variable, the same pattern was observed. Path a replicated the previous result (*B* = 22.240, *p* < 0.001), but path b was non-significant (*B* = −0.001, *SE* = 0.003, *t* = −0.340, *p* = 0.735). The direct effect of experimental condition on log(*k*) was non-significant (*c*’ = 0.110, *SE* = 0.087, *t* = 1.258, *p* = 0.211), as was the total effect (c = 0.087, *SE* = 0.054, *t* = 1.595, *p* = 0.113). The indirect effect was −0.023 (*SE* = 0.068, 95% *CI* [−0.158, 0.113]), with the confidence interval including zero, ruling out statistically significant mediation.

Taken together, the mediation analyses indicate that, although noise exposure induced a significant and large-magnitude increase in cardiac activation, this activation did not operate as a mediating mechanism between experimental condition and decision-making performance in either domain evaluated. The absence of significant mediation is consistent with the findings from the correlational analyses and the ANCOVA, and reinforces the conclusion that the physiological stress response induced by noise was not sufficient to impair decision-making performance under the conditions of the present study. These results are discussed considering theoretical models concerning the conditions under which environmental stress impacts higher-order executive processes.

### 3.8 MCQ response consistency

To evaluate the quality of MCQ responses, an internal consistency index was calculated for each participant following the criterion established by Kirby et al. (1999), whereby a consistency of 75% or above indicates a response pattern coherent with the hyperbolic discounting model. The analysis revealed that 85.9% of Control Group participants and 80.4% of Experimental Group participants obtained consistency indices below this threshold. A Mann–Whitney U test indicated no significant between-group differences in consistency levels (Experimental Group: *M* = 0.552, *SD* = 0.216; Control Group: *M* = 0.560, *SD* = 0.174), *Z* = −0.615, *p* = 0.538, suggesting that noise exposure did not differentially affect the coherence of MCQ responses.

### 3.9 Exploratory analysis: sex differences

To examine whether sex moderated the effects of noise exposure on decision-making performance and subjective stress, exploratory Mann–Whitney U analyses were conducted separately for the Experimental Group and the Control Group, comparing male and female participants on IGT Total Score, log(*k*) MCQ, and Stress change post-pre.

Within the Experimental Group (*n* = 56; 28 men, 28 women), no significant sex differences were found for Stress change (*Z* = −0.423, *p* = 0.673, *r* = 0.057), IGT Total Score (*Z* = −0.395, *p* = 0.693, *r* = 0.053), or log(*k*) MCQ (*Z* = −1.557, *p* = 0.119, *r* = 0.208). Similarly, within the Control Group (*n* = 78; 39 men, 39 women), no significant sex differences emerged for Stress change (*Z* = −1.162, *p* = 0.245, *r* = 0.132), IGT Total Score (*Z* = −0.431, *p* = 0.667, *r* = 0.049), or log(*k*) MCQ (*Z* = −1.599, *p* = 0.110, *r* = 0.181). All effect sizes were trivial to small. These findings indicate that sex did not differentially moderate the response to noise exposure or decision-making performance in this sample, and that the pattern of physiological-cognitive dissociation documented in the primary analyses holds equivalently across sexes.

Taken together, the analyses conducted reveal a consistent pattern across all statistical tests employed. High-intensity ambient noise induced a robust and statistically significant autonomic and subjective stress response, with large to very large effect sizes across all physiological and perceptual indicators. In contrast, none of the decision-making performance variables—in either the IGT or the MCQ—showed significant between-group differences, a pattern that persisted after controlling for baseline covariates (ANCOVA) and was confirmed by the absence of significant mediation of ΔHR on cognitive performance. The dissociation between these two response domains constitutes the central finding of the present study; its implications for theoretical models of environmental stress and executive cognition, as well as its limitations and future research directions, are discussed in the following section.

## 4 Discussion

The results of the present study reveal a pattern of dissociation between the physiological and subjective stress response induced by noise and performance on decision-making tasks. Experimental exposure to high-intensity urban noise (85–95 dB[A]) produced robust autonomic activation, evidenced by significant and large-magnitude increases in heart rate throughout the experimental phase, as well as a marked increase in post-task stress self-perception. However, this acoustic stressor did not impair performance on either decision-making task evaluated—neither risk-based (IGT) nor intertemporal (MCQ)—nor did it operate as a mediating mechanism between experimental condition and cognitive performance. This pattern of dissociation between peripheral physiological activation and cognitive performance constitutes the central finding of the study and warrants detailed discussion considering existing theoretical models and the particularities of the protocol employed.

### 4.1 Noise did not impair decision-making performance (H1)

The first hypothesis was not confirmed: exposure to high-intensity urban noise produced no significant between-group differences in IGT Total Score, individual deck choices, or the MCQ log(*k*) parameter. This null finding is, in itself, theoretically informative and converges with the heterogeneity of results documented in the stress and decision-making literature (Starcke and Brand, 2012, 2016; Haushofer et al., 2013). Several explanatory hypotheses, not mutually exclusive, may account for this absence of effect.

A first explanation points to the neurobiological nature of the stressor. Unlike the Trier Social Stress Test (TSST; Kirschbaum et al., 1993)—which combines social evaluative threat and uncontrollability and generates two- to fourfold cortisol elevations with a salivary peak between 10 and 20 min after the stressor—noise preferentially activates the sympathetic-adrenal-medullary (SAM) axis without necessarily reaching the HPA axis activation threshold required to produce the cortisol levels that impair prefrontal function (Münzel et al., 2018). Cortisol, in interaction with catecholamines, is what deteriorates executive function and biases decisions (Arnsten, 2009, 2015; Kimura et al., 2013). Additionally, Münzel et al. (2014) documented that vegetative activations habituate to noise to a lesser degree than cortical arousals, suggesting a dissociation between peripheral autonomic response and central cognitive impact. The absence of salivary cortisol measurement in the present study precludes direct confirmation of this hypothesis and constitutes a methodological limitation discussed further below.

A second explanation concerns habituation during the task itself. Given that noise was active from room entry and the IGT comprises 100 continuous trials, participants likely experienced progressive psychological habituation to the stressor—a mechanism of noise isolation from conscious awareness that reduces emotional overload in the prefrontal cortex, even as the autonomic response persists (Münzel et al., 2018, 2020). This would explain why HR remained elevated while decision-making performance was unaffected.

The cognitive characteristics of the tasks may also have acted as a protective factor. In the MCQ, stimuli are visual and explicit: participants simultaneously observe amounts and delays, allowing direct comparison without reliance on working memory—a prefrontal resource highly sensitive to stress (Arnsten, 2009). In the IGT, feedback-based learning involves striatal circuits that are paradoxically strengthened—not impaired—by stress-associated catecholaminergic activation (Arnsten, 2015), which may have compensated for any deterioration in deliberative prefrontal processing. Both findings are consistent with the inverted-U model (Yerkes and Dodson, 1908; Arnsten and Goldman-Rakic, 1998): tasks with greater external informational support require higher arousal levels to show performance decrements.

An additional contextual factor is the participants’ possible chronic habituation to noise. University students in Medellín are daily exposed to levels of 75–95 dB(A) on high-activity roads (Zamorano-González et al., 2021; Zannin et al., 2003), comparable to those used in the protocol. Chronic exposure can reduce the cortisol response to homologous stressors without eliminating peripheral sympathetic activation (Münzel et al., 2020), which would explain the HR elevation without observable cognitive impairment—although verification would require cross-cultural comparisons or direct cortisol measurement (Münzel et al., 2018).

Finally, a temporal explanation warrants consideration. The MCQ was administered at the beginning of the experimental phase, immediately after a five-minute adaptation period, possibly before any cortisol response had reached its peak—documented between 15 and 20 min after the stressor in TSST protocols (Kirschbaum et al., 1993). Even if noise had activated the HPA axis, the cognitive effects of cortisol may have manifested at a later point than assessed (Kimura et al., 2013; Duque et al., 2022), a hypothesis consistent with the role of assessment timing as a critical moderator of stress effects on decision-making (Sarmiento et al., 2024b).

### 4.2 Noise induced physiological and subjective stress (H2)

The second hypothesis was broadly confirmed. The Experimental Group exhibited significant and large-magnitude increases in HR at all time points of the experimental phase, with effect sizes ranging from *r* = 0.549 to *r* = 0.856—among the largest reported in laboratory experimental stress studies. The complete autonomic recovery observed during the recovery phase (*p* = 0.641) confirms that the response was transient and specific to stressor exposure, as is characteristic of acute sympathetic responses (Münzel et al., 2018). These findings are consistent with the literature on cardiovascular effects of high-intensity urban noise (Münzel et al., 2014, 2018, 2020, 2025) and confirm the efficacy of the experimental manipulation in inducing an objectively measurable autonomic response. Likewise, the marked increase in post-task stress self-perception in the Experimental Group (*M* = 9.71 out of 10, compared to *M* = 2.92 in the Control Group) confirms that the stressor was subjectively perceived as highly stressful, validating the experimental manipulation at both physiological and psychological levels. Baseline perceived stress as measured by the PSS-14 did not differ between groups, confirming initial equivalence in chronic stress state, and baseline HR likewise did not differ, ruling out pre-existing differences in autonomic reactivity.

### 4.3 HR did not mediate decision-making performance (H3)

The third hypothesis was likewise not confirmed. Correlations between cardiac activation indicators (ΔHR, Mid-task HR) and performance on the IGT and MCQ were null or small and non-significant at all three levels of analysis—total sample, Experimental Group, and Control Group—, and the mediation analysis confirmed that ΔHR did not operate as a transmitting mechanism between experimental condition and decision-making performance in either domain. This finding must be interpreted considering a fundamental methodological distinction: the present study employed HR as an indicator of sympathetic activation, whereas the literature linking autonomic function to decision-making performance typically uses heart rate variability (HRV)—particularly the RMSSD index reflecting vagal tone—as an indicator of autoregulatory capacity (Forte et al., 2019, 2022; Thayer and Lane, 2000). HR and HRV are distinct measures: elevated heart rate can coexist with reduced HRV, and it is precisely the reduction in vagal tone—not elevated HR per se—that is associated with executive impairment according to the Neurovisceral Integration Model (Thayer and Lane, 2000). In this sense, the elevated HR observed in the Experimental Group may reflect sympathetic dominance without necessarily implying an equivalent reduction in parasympathetic tone or prefrontal executive control. The absence of HRV measurement in the present study precludes direct evaluation of this hypothesis and constitutes an additional methodological limitation of theoretical relevance.

### 4.4 Theoretical implications

The findings of the present study contribute to delineating the conditions under which environmental stress impacts decision-making. The observed dissociation between autonomic activation and cognitive performance suggests that not every form of stress that elevates HR is equivalent in its effects on cognition. Arnsten's (2009, 2015) theoretical model predicts prefrontal impairment when catecholamine levels exceed the activation threshold of intracellular signaling cascades that weaken synaptic efficacy—a threshold that may not be reached by acoustic noise in the absence of the self-threat that characterizes psychosocial stressors. Analogously, the somatic marker hypothesis (Bechara and Damasio, 2005) proposes that decisional impairment under stress occurs when bodily signals are disrupted or misinterpreted by the prefrontal-amygdala system; if the processing of these signals remains functional, decisions may remain adaptive even in the presence of elevated autonomic activation. The present findings also enrich the debate on stressor specificity in models of stress and cognition (Starcke and Brand, 2016; Sarmiento et al., 2024b): stressor type, sensory modality, degree of perceived control, timing of assessment, and prior exposure history emerge as critical modulators of the relationship between environmental stress and decision-making.

### 4.5 Practical implications

Despite the absence of effects on decision-making performance, the physiological findings of the present study carry substantial practical implications. The magnitude of the documented autonomic response—with increases of up to 27 bpm in mean HR during the task—indicates that high-intensity urban noise constitutes a first-order cardiovascular stressor that, while not impairing decisions under acute controlled exposure, could have cumulative consequences on cardiovascular health, circadian regulation, and neuroendocrine function under conditions of chronic exposure (Badea, 2025; Münzel et al., 2020, 2025). Meta-analytic evidence further indicates that chronic environmental noise exposure is significantly associated with increased risk of depression and anxiety (RR = 1.29; Hu, 2025), and with neurobiological changes including neuroinflammation and hypothalamic–pituitary–adrenal axis dysregulation that increase vulnerability to mental health disorders (Hahad et al., 2025). From a public health perspective, these findings underscore the need to implement urban noise mitigation strategies in Latin American cities, where acoustic exposure levels consistently exceed WHO-recommended thresholds (World Health Organization, 2018; Zamorano-González et al., 2021). The findings also carry direct implications for the design of occupational, educational, and clinical environments where high-stakes decisions are made under sustained noise exposure—including operating rooms, open-plan offices, emergency services, and urban schools—where noise-induced autonomic stress may accumulate over hours or days even when immediate cognitive performance appears preserved (Hahad et al., 2025; Syndicus et al., 2016). Finally, the ecologically valid acoustic stimulus employed in this study—capturing the full acoustic complexity of a Latin American urban street—supports the translational relevance of these findings for noise policy and urban planning in the Global South.

### 4.6 Limitations

The present study has certain methodological limitations that should be considered when interpreting the results. First, the absence of salivary cortisol measurement precludes determining the degree of HPA axis activation and, therefore, establishing whether the neurobiological conditions for the prefrontal impairment described by Arnsten (2009, 2015) were effectively reached. Second, the design assigned participants to conditions consecutively according to the recruitment period, which introduces the possibility of confounding variables related to the temporal context of evaluation. However, the statistical equivalence between groups across all baseline variables—including baseline HR, PSS-14, age, gender, and years of schooling—mitigates this concern. Third, MCQ consistency was below the threshold established by Kirby et al. (1999) in a substantial proportion of participants. However, given that groups did not differ in consistency, this factor does not differentially bias the results.

An additional limitation concerns the absence of individual noise sensitivity assessment. Noise sensitivity—defined as the degree to which individuals are susceptible to the adverse effects of noise exposure—is a well-documented moderator of both physiological and psychological responses to acoustic stressors (Tiwari et al., 2023), and its absence as a covariate may have obscured individual differences in autonomic reactivity and subjective stress perception between participants. Future studies should incorporate validated noise sensitivity measures, such as the Weinstein Noise Sensitivity Scale, to examine whether individual sensitivity moderates the relationship between noise exposure and cognitive performance. Furthermore, the exclusive reliance on heart rate as the sole psychophysiological indicator, in the absence of HRV and salivary cortisol measurements, limits inferences about vagal tone and HPA axis activation—the neurobiological mechanisms most directly implicated in stress-induced cognitive impairment according to current theoretical models (Arnsten, 2015; Münzel et al., 2018; Sarmiento et al., 2024a). Nevertheless, the random assignment of participants to conditions ensures that individual differences in noise sensitivity were distributed equivalently across groups, minimizing the risk of systematic bias in between-group comparisons. Similarly, while heart rate does not capture vagal tone, it constitutes a widely validated indicator of sympathetic activation (Immanuel et al., 2023), and its use is consistent with the study’s primary focus on SAM-axis responses rather than HPA-axis activation.

Finally, the findings are applicable to healthy young university adults and cannot be directly generalized to populations with greater physiological or psychological vulnerability to stress. Specifically, the results may not extend to older adults, working professionals with distinct stress profiles, or individuals chronically exposed to high-intensity urban noise, who may exhibit different physiological reactivity, cognitive reserve, or habituation patterns in response to acoustic stressors (Münzel et al., 2020; Badea, 2025). Future studies should examine whether the observed physiological-cognitive dissociation replicates across these populations.

### 4.7 Future directions

The present findings point to several lines of future research. The most immediate is replication of the protocol incorporating salivary cortisol measurement at multiple time points—including 15 and 30 min after noise onset—, which would allow characterization of HPA axis activation and examination of whether decisional impairment emerges when assessment is conducted at the cortisol response peak (Kirschbaum et al., 1993; Duque et al., 2022). The incorporation of HRV measures would additionally allow evaluation of the Neurovisceral Integration Model (Thayer and Lane, 2000) with greater methodological precision. Future research could also examine whether the effect of noise on decision-making is moderated by task presentation format—comparing visual and auditory versions of the MCQ—or by chronic noise exposure history in Latin American urban contexts. Finally, the use of more ecologically valid and dynamic decision-making paradigms requiring greater working memory demand under continuous noise could reveal effects not detected with the instruments employed in the present study (Syndicus et al., 2016; Baumgart et al., 2024). Studies including diverse populations—older adults, working professionals, and individuals with histories of chronic occupational noise exposure—would substantially extend the external validity of these findings.

## 5 Conclusion

The present study examined the effect of acute exposure to high-intensity urban noise on risk-based and intertemporal decision-making, finding that, although noise induced a robust autonomic and subjective stress response, it did not impair performance on any of the evaluated tasks. This pattern of physiological-cognitive dissociation enriches theoretical models of environmental stress and cognition, suggesting that peripheral sympathetic activation is not a sufficient condition to impair prefrontal-dependent decision-making processes—at least under conditions of acute, moderate exposure and in a population with possible chronic habituation to urban noise. The findings underscore the importance of characterizing not only the magnitude, but also the neurobiological nature of the stressor, the timing of assessment, and the participant’s exposure history as critical modulators of the relationship between environmental stress and executive cognition (Sarmiento et al., 2024b; Starcke and Brand, 2016). In the context of growing Latin American urbanization and its elevated levels of acoustic pollution, these results represent an initial and empirically grounded contribution toward understanding how the urban soundscape shapes—or does not shape—the quality of our everyday decisions.

## Data Availability

The raw data supporting the conclusions of this article will be made available by the authors, without undue reservation.
