# Supplementary material for: Urban noise-induced stress and decision-making: effects of high-intensity acoustic exposure on risk-based and intertemporal choices
Source: Front Psychol. 2026 Aug 10;17:1899506. doi: 10.3389/fpsyg.2026.1899506 (PMC13500585; doi:10.3389/fpsyg.2026.1899506)
Supplement: Supplementary file 1 [file Table_1.docx]

**Supplementary Material S1** Urban Noise-Induced Stress and Decision-Making: Effects of High-Intensity Acoustic Exposure on Risk-Based and Intertemporal Choices; Ríos-Flórez, J. A., Parra Restrepo, L., & Vivares-Serna, D. S.

**EXPERIMENTAL PROTOCOL**

**Table S1.** *Overview of the experimental protocol phases*

| **Phase** | **Location** | **Duration** | **Key measures** |
| --- | --- | --- | --- |
| 1. Reception | Resting room | ~15 min | BP, Baseline HR |
| 2. Pre-test | Resting room | ~10 min | PSS-14, VAS |
| 3. Tasks | Experimental room | ~45 min | MCQ, IGT, HR ×3 |
| 4. Post-test | Resting room | ~10 min | VAS, Recovery HR, BP |
| 5. Debriefing | Resting room | ~5 min | — |

*Note.* BP: blood pressure; HR: heart rate; PSS-14: 14-item Perceived Stress Scale; VAS: visual analogue scale of stress self-perception (0–10); MCQ: Monetary Choice Questionnaire (27 items); IGT: Iowa Gambling Task (100 trials). Durations are approximate and may vary slightly across participants. HR was recorded at six time points across the protocol; the three recorded during Phase 3 correspond to task onset, mid-task, and task-end.

**PROCEDURE**

**Phase 1. Reception and informed consent.** Participants were received individually in the resting room by one of the research assistants. The study was presented, and the general objectives, procedures, minimal risks, and participant rights —including the right to withdraw without consequences— were explained, after which informed consent was obtained. While the participant remained seated and silent, blood pressure was measured using a digital sphygmomanometer, repeated up to three times at two-minute intervals to ensure that participants began the evaluation tasks in a verified cardiovascular resting state. Subsequently, the first baseline HR measurement was recorded (initial rest, mean HR over one minute), providing an individual baseline reference value for evaluating autonomic changes throughout the protocol. The baseline HR measure was obtained as the mean of three consecutive one-minute recordings, separated by one-minute intervals, with the participant seated and at rest.

**Phase 2. Pre-test (Perceived Stress Scale).** In the resting room, prior to entering the experimental room, the PSS-14, and the visual analogue scale of stress self-perception (0–10) were administered to all participants. This pre-experimental measure of perceived stress was intended to control for interindividual differences in baseline stress state and to verify initial equivalence between groups before the experimental manipulation. Once the questionnaire was completed, the participant was escorted to the experimental room, where the research assistants and evaluation equipment were located.

**Phase 3. Decision-making tasks and physiological recording.** Upon entering the experimental room, a five-minute adaptation period was conducted, at the end of which the second HR measurement was recorded. Fifteen seconds after task onset, the third HR measurement was recorded. Tasks were administered in the following order: first the Monetary Choice Questionnaire (MCQ: 27 items) and then the Iowa Gambling Task (IGT: 100 trials). The fourth HR measurement was recorded at the midpoint of the IGT, and the fifth upon completion of the last task. For participants in the Experimental Group, the urban ambient noise audio (85–95 dB) was active from the moment of entry into the experimental room until the end of the IGT. Control Group participants completed the same tasks under identical physical conditions but in silence.

Throughout Phase 3, participants were attended by two trained research assistants who remained in the experimental room for the entire session. To protect their auditory integrity and minimize stress derived from prolonged noise exposure, the assistants used foam earplugs, which attenuate perceived sound intensity without impeding verbal communication with the participant or observation of their choices. Both the IGT and the MCQ were administered in physical format using printed cards and manipulable materials, to facilitate direct participant interaction with the stimuli and ensure task comprehension in the noise context. Participant responses and choices were recorded in real time by the evaluators using a standardized digital recording system, ensuring data fidelity, feedback, and traceability.

**Phase 4. Post-test and recovery.** Upon completion of the tasks, participants were escorted back to the resting room, where the visual analogue scale of stress self-perception (0–10) was administered as a post-experimental measure to evaluate the effect of the manipulation on perceived stress and to calculate a change score relative to the pre-experimental measure. After five minutes of rest, the sixth and final HR measurement was recorded (recovery). Additionally, a second blood pressure measurement was taken, repeated up to three times at two-minute intervals, to verify the participant's return to a cardiovascular baseline state and ensure that participation concluded under calm conditions. These measures allow evaluation of the speed of autonomic recovery following stressor and task exposure.

**Phase 5. Closure and debriefing.** Once the recovery recording was completed, the research assistants closed the protocol, thanked the participant, and proceeded with the debriefing: a complete explanation of the actual study objectives, the experimental nature of the acoustic manipulation, and the relevance of the findings for understanding the effects of the urban environment on behavior. Experimental Group participants received information about the temporary nature and intensity levels of the acoustic stimuli used, confirming the absence of permanent auditory damage given the exposure duration (approximately 20–30 minutes) within the intensity range employed. Questions were answered and contact information was provided for any follow-up inquiries.
